# Distribution and effects of the muscarinic receptor subtypes in the primary visual cortex

**DOI:** 10.3389/fnsyn.2015.00010

**Published:** 2015-06-19

**Authors:** Marianne Groleau, Jun Il Kang, Frédéric Huppé-Gourgues, Elvire Vaucher

**Affiliations:** Laboratoire de Neurobiologie de la Cognition Visuelle, École d’Optométrie, Université de MontréalMontréal, QC, Canada

**Keywords:** acetylcholine, attention, basal forebrain, cholinergic system, GABAergic interneurons, muscarinic transmission, visual cortex, visual learning

## Abstract

Muscarinic cholinergic receptors modulate the activity and plasticity of the visual cortex. Muscarinic receptors are divided into five subtypes that are not homogeneously distributed throughout the cortical layers and cells types. This distribution results in complex action of the muscarinic receptors in the integration of visual stimuli. Selective activation of the different subtypes can either strengthen or weaken cortical connectivity (e.g., thalamocortical vs. corticocortical), i.e., it can influence the processing of certain stimuli over others. Moreover, muscarinic receptors differentially modulate some functional properties of neurons during experience-dependent activity and cognitive processes and they contribute to the fine-tuning of visual processing. These functions are involved in the mechanisms of attention, maturation and learning in the visual cortex. This minireview describes the anatomo-functional aspects of muscarinic modulation of the primary visual cortex’s (V1) microcircuitry.

## Introduction

Acetylcholine (ACh) is released in the primary visual cortex (V1) by visual stimulation, especially by novel stimuli (Collier and Mitchell, 1966; Laplante et al., 2005) and attentional demand (Herrero et al., 2008). The cholinergic innervation of the cortex originates from the basal forebrain neurons through topographical projections. Specifically, V1 receives cholinergic projections from the horizontal limb of the diagonal band of Broca (Gaykema et al., 1990; Laplante et al., 2005). In V1, ACh modulates the responses of cortical neurons to visual or cortico-cortical inputs through two receptor families, the metabotropic muscarinic receptors (mAChRs) and the ionotropic nicotinic receptors (nAChRs; Prusky et al., 1987; Volpicelli and Levey, 2004; Disney et al., 2007; Thiele, 2013). These receptors are located on axons originating from thalamic, cortical or basalocortical fibers as well as on pyramidal excitatory neurons and inhibitory GABAergic interneurons (Zilles et al., 1989; Mrzljak et al., 1993; Hashimoto et al., 1994; Thiele, 2013). They are found in each level of the V1 cortical circuitry, i.e., the recipient layer of the thalamic projections, in layer IV neurons and their lateral projections, and throughout the vertical intracortical connections that convey the information to supragranular (I, II/III) and infragranular (V, VI) layers (Burkhalter, 1989; Van Hooser, 2007).

The V1 microcircuitry, whose connectivity is organized vertically and horizontally, provides an anatomical substrate for the receptive field—binocularity (Dräger and Olsen, 1980; Grieve, 2005) or ocular dominance (LeVay et al., 1978; Cynader et al., 1987)—and for the selective properties of the neurons—orientation (Grinvald et al., 1986), direction (Shmuel and Grinvald, 1996; DeAngelis et al., 1999) and contrast preference (Levitt and Lund, 1997), for example. Each functional property of the neuron results from the sum and diversity of the connections it receives and might be adapted according to the strength of the inputs received. The strength of the neuronal response further determines the transmission and processing of the stimulus in higher cognitive cortical areas. V1 is thus the first cortical step of the integration of complex visual stimuli. Its modulation by ACh is then important for the selection of specific stimuli from the visual field and the elaboration of fine visual conscious perception.

In this mini review, we discuss how muscarinic transmission plays a key role in neuronal transmission, synaptic strength and the interaction between excitatory and inhibitory neurons. These mechanisms lead to the reinforcement of particular neuronal connections and contribute to the processes of memory, perceptual learning and attention but also to the maturation and the fine-tuning of the visual cortex.

## Muscarinic Receptors’ Organization in the Primary Visual Cortex

In the neonatal and adult cortices, the five subtypes of mAChRs (M1–M5) are present in both pre- and postsynaptic positions (Wess, 2003; Krnjević, 2004). The terms pre- and postsynaptic are used here to identify the neuronal location of the receptors even though the cholinergic system acts in the cerebral cortex mostly by diffuse transmission rather than synaptic transmission (Umbriaco et al., 1994; Descarries et al., 1997) except in layer V, where the synaptic density on cholinergic terminals is particularly rich (Avendano et al., 1996; Turrini et al., 2001). Depending on the species, the density of each subtype of mAChR differs across the cortical layers (I–VI; Gu, 2003). The species-selective immunocytochemical detection of the different subtypes of mAChRs may, however, vary due to the poor specificity of the antibodies, especially in rodents (Jositsch et al., 2009). Many studies have thus used binding or mRNA expression of the mAChRs to localize them within the cortical microcircuitry. In the rodent’s visual cortex, the subtypes M1 and M2 predominate. In humans (and primates), the subtypes M1, M2 and M4 prevail (Flynn et al., 1995).

**Figure 1:**
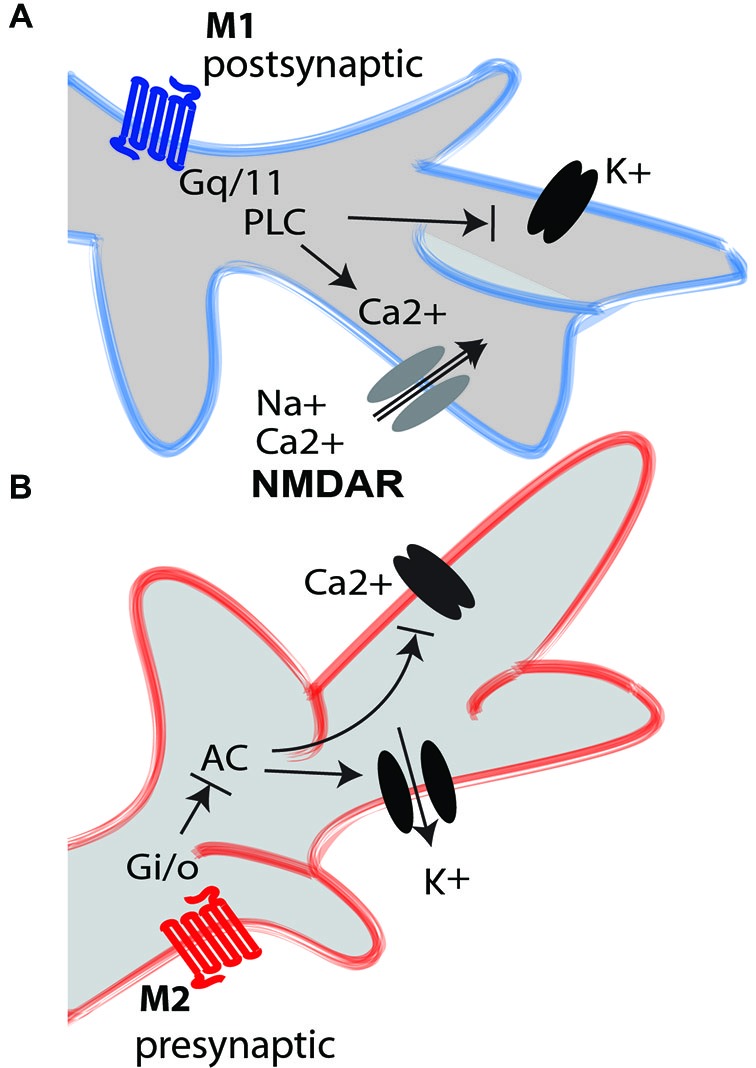
**M1 and M2 mAChRs intracellular mediation. (A)** The activation of the M1 excitatory mAChR (blue) triggers the G_q/11_ G-protein, which activates phospholipase C (PLC). This induces depolarization of the neuronal element by closing different K^+^ channels, including voltage-gated channels and leaky channels, and by activating calcium channels that increase the intracellular concentration of Ca^2+^ from the intracellular stores. The M1 receptor induces long-term potentiation-like effects in glutamatergic neurons through interaction with NMDA receptors (NMDARs). The M1 receptors are mainly postsynaptic, although they are also found on some glutamatergic axon terminals. **(B)** The activation of the M2 inhibitory mAChR (red) triggers the G_i/o_ G-coupled protein, which inhibits adenylate cyclase (AC). This closes the Ca^2+^ voltage-gated channel and opens the K^+^ channel to hyperpolarize the neuron. The M2 receptors are mainly presynaptic, although they are also found on some GABAergic interneurons.

**Figure 2:**
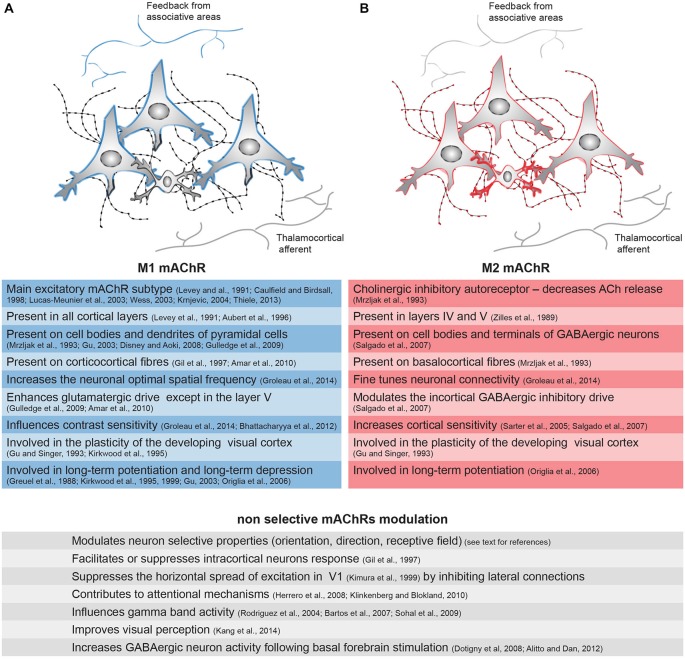
**Distribution of the mAChRs on the inhibitory and excitatory cells of the cortical microcircuitry and their associated functions**. Integration of the information within V1 is mediated through the vertical and horizontal connections between excitatory neurons (large cells) and inhibitory interneurons (small cells). The cortical connections originating from associative areas are represented on the top, and the thalamocortical afferents are represented on the bottom. The cholinergic fibers and their varicosities (swellings) are represented on the back. **(A)** The M1 receptor (blue) is present on the cell bodies and dendrites of pyramidal cells in V1 as well as on the long range cortical connections from associative areas. **(B)** The M2 mAChR (red) is present on the inhibitory interneurons in V1 and also on cholinergic fibers and some pyramidal cells. The thicker the colored line is, the higher the expression of the receptor is. Principal functions of these specific receptors or of the sum of all mAChRs—demonstrated by non-selective agonists or antagonists—are represented in the left (M1 mAChR, blue), right (M2 mAChR, red) and bottom (undifferentiated action of mAChRs) lines.

The M1, M3 and M5 subtypes are mainly post-synaptic and lead to an increase in the intracellular Ca^2+^ concentration by activating phospholipase C (PLC; Figure 1A). These receptors are coupled with Gαq/11 G-proteins. In the cerebral cortex, the M1 subtype, the main excitatory mAChR subtype (Levey et al., 1991; Caulfield and Birdsall, 1998; Lucas-Meunier et al., 2003; Wess, 2003; Krnjević, 2004; Thiele, 2013), appears to be present mainly in layers II/III and VI, but it is found in all the cortical layers (Levey et al., 1991; Aubert et al., 1996; Vaucher et al., 2002; Roberts et al., 2005). In rats, M1 mAChRs represent almost 40% of the total mAChRs (Levey et al., 1991), and in the human occipital cortex, they represent nearly 35% (Flynn et al., 1995). This subtype is found essentially on the cell bodies and dendrites of postsynaptic pyramidal cells (Mrzljak et al., 1993; Gu, 2003; Gulledge et al., 2009; Figure 2A). However, in the primate’s visual cortex, the M1 mAChR seems to be largely expressed on GABAergic interneurons (Disney et al., 2006). M1 is also found on the cortico-cortical fibers, where it plays an inhibitory role by reducing excitatory transmission across horizontal as well as long-range cortico-cortical connections (Amar et al., 2010). The M3 subtype is located on the rat intracortical cell bodies and dendrites at a postsynaptic level, but it is virtually not detected in V1 by immunocytochemistry (Levey et al., 1994). In spite of this, the M3 receptor appears to be involved in several functions of the rodent’s V1 (see other sections), and it is expressed in GABAergic interneurons, where it enhances the transmission of γ-Aminobutyric acid (GABA; Amar et al., 2010). The M5 subtype is found on endothelial cells and only small number is found in the rodent’s (Elhusseiny and Hamel, 2000) and human’s visual cortex (Flynn et al., 1995). The M5 subtype has a major function in cortical perfusion.

The M2 and M4 subtypes are found mostly at the presynaptic level, extending the opening of potassium channels by reducing the intracellular concentration of cAMP (Figure 1B). They are coupled to Gα_i/o_ G-protein, inhibiting adenylyl cyclase (Caulfield and Birdsall, 1998; Wess, 2003). These subtypes appear to have an inhibitory function. Among the presynaptic receptors in the rodent and human visual cortex, the M2 receptor is very abundant and the M4 subtype is less prevalent (Flynn et al., 1995; Zhang et al., 2002). The M2 subtype is mainly found in layer IV (thalamic recipient) and layer V in the rat’s V1 (Zilles et al., 1989), but its distribution in the cortical layers, however, varies depending on the species (Gu, 2003). Its expression is up to 36% of the total mAChRs in the primate’s V1 (Flynn et al., 1995). At the cholinergic terminals, the M2 subtype is the main inhibitory autoreceptor (Mrzljak et al., 1993; Figure 2B) and it decreases the release of ACh, thereby controlling extracellular levels of ACh by negative feedback (Rouse et al., 1999; Douglas et al., 2001; Bymaster et al., 2003). On GABAergic terminals, M2 activation inhibits the release of GABA (Salgado et al., 2007). Although predominantly presynaptic, M2 and M4 receptors are also present on the cell bodies of GABAergic interneurons in layers II/III and IV (Volpicelli and Levey, 2004)—representing 29% of the GABAergic cells in the primate (Disney and Aoki, 2008)—and on pyramidal cells (Mash and Potter, 1986; Kimura and Baughman, 1997), where its activation inhibits excitatory conductance (Amar et al., 2010).

## Muscarinic Influence on Visual Processing in V1

The action of ACh on both pre- and postsynaptic mAChRs results in improved sensory coding of novel and trained visual stimuli (Kang et al., 2014). This change in neuron properties is due to improved neuronal sensitivity resulting from a change in membrane conductance, synaptic strength or connectivity with adjacent neurons and long-range cortical projections. The M1 and M3 subunits seem to have a strong influence on neuronal sensitivity because the optimal spatial frequency of the neuronal population is decreased and the contrast sensitivity is increased in M1/M3-KO mice (Groleau et al., 2014).

ACh has been shown to influence the response of V1 neurons in terms of intensity (Bröcher et al., 1992; Lewandowski et al., 1993; Gil et al., 1997; Kimura et al., 1999; Kirkwood et al., 1999; Kuczewski et al., 2005; Levy et al., 2006; Thiel, 2007; Dotigny et al., 2008; Kang and Vaucher, 2009; Pinto et al., 2013; Soma et al., 2013a,b,c), preferred responses (Murphy and Sillito, 1991; Roberts et al., 2005; Thiel, 2007) and receptive field properties (Herrero et al., 2008; Thiel and Fink, 2008). ACh executes an action by controlling the gain of the neuron response (Soma et al., 2012, 2013a). For example, ACh increases the gain of the visual response to contrast (Bhattacharyya et al., 2013; Soma et al., 2013a) or orientation selectivity (Zinke et al., 2006). These effects might be due to the facilitation of the depolarization of glutamatergic neurons in response to visual input (Figures 1, 2) due to the increased concentration of Ca^2+^ associated with NMDA receptor-gated conductance (Kirkwood et al., 1999) or the reduction of membrane K+ conductance (Thiele, 2013), both potentiated by the muscarinic receptors. The M1 mAChR also amplifies the spiny stellate cell/pyramidal cell response through a postsynaptic intracellular pathway (Gu, 2003), but inhibition through the M4 mAChR has also been observed on spiny neurons in the somatosensory cortex (Eggermann and Feldmeyer, 2009). M2 receptor activation of GABAergic perisomatic terminals (Figures 1, 2) inhibits the release of GABA, causing an increase in the cortical sensitivity of glutamatergic neurons (Sarter and Parikh, 2005; Sarter et al., 2005; Salgado et al., 2007). The M2 subtype, which is largely found on GABAergic cells in rodents, plays a strong role in the modulation of the intracortical GABAergic inhibitory drive.

The amplification of the neuronal response to a certain stimulus could also be due to the depression of the neural response of adjacent neurons that have distinct receptive field and selective properties. By acting on horizontal connections, ACh might thereby modulate the weight of a selective stimulus. In humans, an increase in extracellular ACh levels following the administration of donepezil (an inhibitor of the cholinesterase inhibitor) reduces the horizontal spread of the excitatory response following visual stimulation. This could result from a reduction in the size of the excitatory receptive field by ACh due to the depression of the lateral connectivity (Silver et al., 2008). The reduction of the spread of lateral excitation (Kimura et al., 1999) and neuron depression (Kimura and Baughman, 1997; Soma et al., 2013b) following ACh administration is also shown in rodents. It is, however, possible that the cholinergic system not only inhibits the lateral competition but also strengthens the connectivity for a trained orientation, thereby increasing the number of responding neurons to this trained orientation (Kang et al., 2014). In primates, it has been suggested that the lateral connections between similarly tuned neurons are reinforced by cholinergic stimulation (Ramalingam et al., 2013). Such a change increases the cortical response (Frenkel et al., 2006), enhances the sensitivity of trained visual stimulus (Matthews et al., 1999) and thus facilitates the discrimination from the background (Jehee et al., 2012).

An alternate action of the mAChRs in the increase of the neuron sensitivity of the afferent visual inputs is the increase in the long-term responsiveness of the neuron, leading to an acquired change of its functional property. The action mechanism of ACh strongly resembles long-term potentiation (Gu, 2003; Kang and Vaucher, 2009; Rodriguez et al., 2010; Kang et al., 2014) and heterosynaptic facilitation. When repetitive visual stimulation of sub-optimal orientation is paired with the application of ACh, the responses of neurons become stronger and more long-lasting at the expense of a diminishing response to the previous optimal orientation (Greuel et al., 1988; Kang et al., 2014). Moreover, coupling visual stimulation with cholinergic stimulation induces long-lasting increases in cortical responsiveness and improved visual acuity (Dringenberg et al., 2007; Kang and Vaucher, 2009; Kang et al., 2014) relative to NMDA-dependent mechanisms. The joint action of ACh on both GABAergic and glutamatergic neurons also compromises the excitation-inhibition balance (Amar et al., 2010). This would induce cortical plasticity (Arckens et al., 2000; Hensch and Fagiolini, 2005; Benali et al., 2008; Mainardi et al., 2009; Sale et al., 2010).

## Muscarinic Influence on the Development and Maturation of the Visual Cortex

The above muscarinic contribution to the tuning of the receptive field and preferred properties of V1 neurons has a potent role in the maturation and fine-tuning of the visual cortex. The retinotopic organization of V1 is established during embryogenesis, and the properties of the neurons are acquired and refined during the post-natal period with visual experience, especially during the critical period. The critical period is thus an important time in the formation of synapses and pruning (Consonni et al., 2009) and for synaptic plasticity, which strengthens and stabilizes the neural connections.

It has been shown that the cholinergic system is essential during embryogenesis, although the amount of M1, M2 and M3 receptors is very small at the end of the rat prenatal period compared with the adult animal. The cholinergic innervation in V1 is settled at the end of the first postnatal week, and a robust cholinergic staining is visible at P8 (Mechawar and Descarries, 2001). It is similar to the adult cholinergic innervation of the cortex at the end of the second postnatal week (Mechawar and Descarries, 2001). The cholinergic receptors are present in the cortex before the beginning of the critical period, which starts at the end of the third postnatal week (Fagiolini et al., 1994). Between weeks 3 and 5, M1 and M3 levels reach the levels found in the mature animal, while it is not until week 5 that the M2 receptor level reaches that found in the adult (Aubert et al., 1996). Thus, the level of muscarinic expression fits well with the acquisition of the functional properties of the V1 neurons and the establishment of the functional maps. In agreement with a role of the mAChRs in the maturation of the visual cortex rather than development by itself, we recently showed that the gross retinotopic map was virtually unaffected by diverse mAChR subtypes’ deletion (Groleau et al., 2014). However, there was altered neuronal connectivity in adult M2/M4-KO mice as visualized using intrinsic signal optical imaging. In these animals, the spatial representation of the visual field was not smooth as it was in control mice, but rather it was stepwise, suggesting a lack of fine-tuning of the retinotopic map. M1/M3 deletion resulted in an alteration of the neurons’ sensitivity. Therefore, different mAChRs or combinations thereof can modulate visual properties during the establishment of visual functions (Groleau et al., 2014).

In rodents, a basal forebrain lesion during the critical period transiently affects the ocular dominance of the visual cortical neurons, i.e., the preference response of the neuron to input of one eye over the other. In basal forebrain lesioned animals, an altered ocular dominance toward the contralateral eye is observed. However, at the end of the critical period, a cholinergic deafferentation does not alter ocular dominance (Siciliano et al., 1997). Immunolesion of the cholinergic fibers affects the mRNA expression of the M1 and M2 mAChR subtypes as measured by RT-PCR in young animals (Kuczewski et al., 2005), suggesting the involvement of these mAChR subtypes in the plasticity of the developing visual cortex. At the receptor level, the M1 subtype, but not the M2 subtype, is involved in ocular dominance because its blockade prevents the shift of ocular dominance (Gu and Singer, 1993).

The stabilization of the neuronal connections during maturation happens through synaptic plasticity, i.e., long-term potentiation and long-term depression. In the cortex, long-term potentiation is strongly active during the critical period and experience-dependent plasticity (Crair and Malenka, 1995; Kirkwood et al., 1995). The involvement of mAChRs in critical period plasticity has been demonstrated through *in vitro* electrical stimulation. Long-term depression is dependent on the M1 receptor in layers II/III of the V1 in young rats (3–4 weeks). In adults, long-term depression also depends on the M3 receptor in addition to the M1 subtype (McCoy and McMahon, 2010). When the visual cortex was stimulated through a 100 Hz tetanic stimulation, long-term potentiation was recorded in the cortex of young M1/M3-KO, but not in M2/M4-KO, mice. Conversely, low frequency stimulation produced expected long-term depression in M2/M4-KO mice while long-term potentiation was recorded in M1/M3-KO mice. Thus, it appears that various subtypes of mAChRs regulate distinct forms of long-term synaptic plasticity (Origlia et al., 2006).

## Muscarinic Influence on Visual Cognition

In adults, the effect of ACh on neuron sensitivity and the long-lasting enhancement of neuronal responses contribute to the processes of attention and perceptual learning. Indeed, the intensity of the response of V1 cells to a particular stimulus as well as the number of cells responding to the stimulus determine the weight for further processing of this stimulus in higher-level cortical areas, i.e., enhanced or depressed visual processing. In learning and experience-dependent acquisition of new visual abilities, the response selectivities of V1 neurons are changed (Froemke et al., 2007), as are neural connections, with an increased number of synaptic contacts or the formation of new neurons (Majewska and Sur, 2003; Hofer et al., 2009; Yamahachi et al., 2009). The synapse strength of V1 neurons is adjusted by long-term potentiation or depression, which is dependent on N-Methyl-D-aspartate receptor (NMDAR; Quinlan et al., 2004; de Marchena et al., 2008; Kang and Vaucher, 2009) and induces a persistent increase of cortical responsiveness to a particular stimulus. The synchronization of a large number of neurons firing rises to macroscopic oscillations, which change cortical activity.

Oscillation in gamma frequency is suggested to reflect cognitive activity, such as sensory perception (Cardin et al., 2009), attention (Fries, 2009) and learning (Paik and Glaser, 2010; Headley and Weinberger, 2011). Previous studies have demonstrated that cholinergic stimulation could increase gamma band activity (Rodriguez et al., 2004), and this can enhance visual encoding (Goard and Dan, 2009) or contrast sensitivity (Bhattacharyya et al., 2013). Specifically, the muscarinic influence on gamma band activity might be due to its action on GABAergic cells, which are also involved in gamma oscillations (Bartos et al., 2007; Sohal et al., 2009).

A number of studies have shown that lesion or blockade of the cholinergic system with antagonist injection in the primary sensory cortex could significantly reduce attentional task performance (Klinkenberg and Blokland, 2010). Different studies have shown that ACh could increase either pre- or postsynaptic responses via mAChR (Gil et al., 1997; Oldford and Castro-Alamancos, 2003). Such variation enables the cholinergic system to amplify relevant information at the expense of unreliable information, which is consistent with the function of attention (Briggs et al., 2013). A voluntary focus on a stimulus observed in top down attention originates from long range cortico-cortical connections from associative areas and the prefrontal cortex compared with bottom up attention reaching layer IV from thalamic afferents. Bottom up attention does not seem to be altered by the cholinergic system (Rokem and Silver, 2010), but sustained attention is altered by it. For example, cholinergic-dependent visual attention also results in modulating the size of the cortical receptive field. Focused attention within the receptive field will result in a decrease of its size, whereas attention paid right next to the receptive field will result in an increase in its size (Anton-Erxleben et al., 2009). Scopolamine, a non-specific mAChR antagonist, has been shown to disrupt the attentional mechanism at various levels (Klinkenberg and Blokland, 2010). Similarly, in V1, voluntary visual attention is suppressed by the blockade of mAChR (Herrero et al., 2008).

Compared with attention, which emphasizes the upcoming information, perceptual learning is a long-term process that improves behavioral performance after repetitive training. Recent studies have demonstrated that cholinergic innervation in V1 facilitates perceptual learning in rodents (Kang et al., 2014) and in humans (Rokem and Silver, 2010). Cholinergic activation during a visual task seems to increase the cortical response, resulting in an enhancement of visual capacity. An increase in the cortical response to the trained stimulus suggests an increase in the number of neurons encoding stimulus properties (Frenkel et al., 2006) and the efficiency of the neuronal transmission between neurons (Gilbert and Li, 2012). mAChR-induced long-term modulation could thus change the efficiency of selective neuronal networks for this trained stimulus through the modulation of lateral connectivity and the enhancement of some feed-forward inputs. For example, a visual stimulus with the preferred orientation presented outside of the classic receptive field normally suppresses the neuronal visual response. However, after a perceptual learning task, the neuronal response can be enhanced (Kapadia et al., 2000) by this stimulus. Overall, a long-term increase in cortical neurons’ activation could be due to mAChR strengthening the lateral connectivity between similarly tuned neurons, thereby changing the orientation index or the receptive field size.

## Conclusion

Muscarinic transmission influences visual processing by facilitating or depressing neuronal responses to specific stimuli and by modulating lateral connections’ strength and neuronal synchronization. This effect is primarily mediated through M1 and M2 mAChRs, the predominant muscarinic subtypes in V1, at least in rodents. These effects result in fine-tuning of the neuronal and network properties during maturation, attention and perceptual learning.

## Conflict of Interest Statement

The authors declare that the research was conducted in the absence of any commercial or financial relationships that could be construed as a potential conflict of interest.

## Abbreviations

ACh: acetylcholine
GABA: γ-Aminobutyric acid
KO: knock-out
mAChRs: muscarinic acetylcholine receptors
M1, M2, M3, M4, M5: muscarinic receptor subtypes 1–5
nAChRs: nicotinic acetylcholine receptors
NMDAR: N-Methyl-D-aspartate receptor
V1: primary visual cortex.
